# A novel nomogram can predict pathological T3a upstaged from clinical T1a in localized renal cell carcinoma

**DOI:** 10.1590/S1677-5538.IBJU.2021.0859

**Published:** 2022-05-18

**Authors:** Chuanzhen Cao, Xiangpeng Kang, Bingqing Shang, Jianzhong Shou, Hongzhe Shi, Weixing Jiang, Ruiyang Xie, Jin Zhang, Lianyu Zhang, Shan Zheng, Xingang Bi, Changling Li, Jianhui Ma

**Affiliations:** 1 Chinese Academy of Medical Sciences and Peking Union Medical College Cancer Hospital National Clinical Research Center for Cancer Beijing China Department of Urology, National Cancer Center, National Clinical Research Center for Cancer, Cancer Hospital, Chinese Academy of Medical Sciences and Peking Union Medical College, Beijing, China;; 2 Chinese Academy of Medical Sciences and Peking Union Medical College Cancer Hospital National Clinical Research Center for Cancer Beijing China Department of Imaging, National Cancer Center, National Clinical Research Center for Cancer, Cancer Hospital, Chinese Academy of Medical Sciences and Peking Union Medical College, Beijing China;; 3 Chinese Academy of Medical Sciences and Peking Union Medical College Cancer Hospital National Clinical Research Center for Cancer Beijing China Department of Pathology, National Cancer Center, National Clinical Research Center for Cancer, Cancer Hospital, Chinese Academy of Medical Sciences and Peking Union Medical College, Beijing China

**Keywords:** Carcinoma, Renal Cell, Prognosis, Nomograms

## Abstract

**Hypothesis:**

Nomogram can be built to predict the pathological T3a upstaging from clinical T1a in patients with localized renal cell carcinoma before surgery.

**Purpose:**

Renal cell carcinoma (RCC) patients with clinical T1a (cT1a) disease who are upstaged to pathological T3a (pT3a) have reduced survivals after partial nephrectomy. We aimed to develop a nomogram-based model predicting pT3a upstaging in RCC patients with preoperative cT1a based on multiple preoperative blood indexes and oncological characteristics.

**Materials and Methods:**

Between 2010 and 2019, 510 patients with cT1a RCC were individually matched according to pT3a upstaging and pathological T1a (pT1a) at a 1:4 ratio using clinicopathologic features. Least absolute shrinkage and selection operator regression analysis was used to identify the most important risk factor from 40 peripheral blood indicators, and a predictive model was established. Multivariate logistic regression analysis was performed with the screened blood parameters and clinical data to identify significant variables. Harrell’s concordance index (C-index) was applied to evaluate the accuracy of the model for predicting pT3a upstaging in patients with cT1a RCC.

**Results:**

Out of 40 blood indexes, the top ranked predictor was fibrinogen (FIB). Age, the ratio of the tumor maximum and minimum diameter (ROD), FIB, and tumor size were all independent risk factors for pT3a upstaging in multivariate analysis. A predictive ARFS model (Age, ROD, FIB, tumor Size) was established, and the C-index was 0.756 (95% CI, 0.681-0.831) and 0.712 (95% CI, 0.638-0.785) in the training and validation cohorts, respectively.

**Conclusions:**

Older age, higher ROD, increased FIB level, and larger tumor size were independent risk factors for upstaging. The ARFS model has a high prediction efficiency for pT3a upstaging in patients with cT1a RCC.

## INTRODUCTION

Renal cell carcinoma (RCC) is the third most common urological tumor. Approximately 403,262 new cases of renal cell carcinoma were diagnosed and 175,098 patients died worldwide in 2018, and the incidence rate and mortality continue to increase (1). According to the American Joint Committee on Cancer stage (the 8th AJCC stage), localized T1 RCC (stage I) is classified only according to tumor size (T1a ≤ 4 cm, and 4cm < T1b ≤ 7 cm), while T3a (stage III) is classified according to the presence of peripheral fat invasion, renal sinus fat infiltration, pelvicalyceal system invasion or renal vein extension regardless of the tumor size. The five-year survival rate for stage Ⅰ to stage Ⅲ RCC is reduced from 95% to 60% (2).

For clinical T1 (cT1) tumors, partial nephrectomy (PN) is the preferred treatment, especially for clinical T1a (cT1a) (3). PN can better protect kidney function, reduce the occurrence of chronic kidney disease, and decrease cardiovascular risk (4, 5). Meanwhile, radical nephrectomy (RN) is recommended for clinical T3a disease, excluding patients with a solitary kidney, inadequate contralateral renal function, and bilateral synchronous RCC (6). However, there is a risk of upstaging to pathological T3a (pT3a) when performing PN for cT1a as identifying fat invasion by preoperative imaging can be challenging. Existing studies have reported that 4.4% - 13.3% of cT1 tumors were upstaged to pT3a after surgery (7-10). Non-clear cell RCC had a much higher likelihood of pseudocapsule or fat invasion (11). Upstaging to pT3a could jeopardize oncological outcomes, probably due to positive surgical margins or other factors (9, 12, 13). Although RN is recommended for patients with T3, whether RN is better than PN in these upstaging cases remains debated (14). Preoperative prediction of cT1 upstaging could assist urologists in determining the surgical strategy. Previous studies have suggested that preoperative risk factors such as age, tumor size, hilar location, mean platelet volume (MPV), and serum aspartate aminotransferase (AST)/alanine aminotransferase (ALT) ratio may be related to cT1 upstaging to pT3a (15-18), but there is a lack of prediction indicators or models. Our work aims to establish a nomogram that can predict pathological T3a upstaging from clinical T1a disease in patients with localized renal cell carcinoma before surgery.

In this study, we reviewed RCC patients with cT1a upstaging to pT3a and screened four risk factors: age, the ratio of the tumor maximum and minimum diameter (ROD), fibrinogen (FIB) and tumor size. Among them, ROD is a unique tumor morphology indicator that we introduced in previous research (19). We established and validated the predictive ARFS model based on the four above risk factors, which is beneficial to guide the choice of surgical methods for patients with cT1a.

## MATERIALS AND METHODS

### Patients

The study protocol was approved by the Ethics Committee of the Cancer Institute and Hospital of the CAMS (approval number: 20/245-2441). Localized RCC patients with pT3a upstaging from cT1a between January 2010 and December 2019 at the Cancer Institute and Hospital of the Chinese Academy of Medical Sciences (CAMS) were reviewed. The inclusion criteria of the study were as follows: (1) no primary cancer of any other organs before RN or PN; (2) no chronic inflammatory allergic disease (avoiding interfering blood indexes, such as CRP and immunoglobulin, during screening peripheral blood indicators); (3) no history of anticoagulants use, such as for cardiovascular or cerebrovascular thrombosis; (4) exact pathological diagnosis of RCC; (5) complete resection of the tumor, which was defined as a negative surgical margin; (6) complete clinicopathological characteristics; (7) preoperative assessment of the diameter of the renal tumor by contrast-enhanced computer tomography (CT) or magnetic resonance (MR); and (8) no evidence of extrarenal metastasis. Additional RCC patients with a final pathological diagnosis of T1a (pT1a) were individually matched at a 1:4 ratio. The clinical T stage was assessed with contrast-enhanced CT or MRI. All patients signed informed consents in each medical record.

### Clinicopathological data

Clinicopathologic parameters such as age, sex, clinical and histopathological characteristics, and preoperative peripheral blood indexes were investigated retrospectively. The dimensions of the primary tumor were measured in three planes (coronal, sagittal, and axial), and the maximum diameters of these planes were measured separately by two radiologists. The three maximum diameters in the three planes were named the maximum diameter, submaximum diameter, and minimum diameter according to the value. The ROD was determined as the ratio of the maximum diameter to the minimum diameter. Pathological staging was evaluated according to the 8th AJCC stage. Additionally, peripheral blood samples were obtained 10 days (range, 7-14) before the operation in our center.

### Statistical Analysis

The dataset was split into training and validation cohorts with repeated random sampling until there was no significant difference between the two cohorts with respect to all variables. The tumor and blood indexes out of 40 blood indexes were selected by least absolute shrinkage and selection operator (LASSO) regression (R software and ‘glmnet’ package). Then, multivariate logistic regression analysis was performed with the screened blood parameters and clinical data to identify significant variables. We evaluated the prognostic accuracy of the risk model using Harrell’s concordance index (C-index), which is appropriate for censored data. Both the multivariable logistic regression model and the C-index were completed with R version 3.6.2, and the mean C-index was calculated using Stata 14.0 (Stata Corp. Texas, USA). The P value was calculated using Welch’s t test for continuous variables and χ2 test or Fisher’s exact test for categorical variables. All statistical tests were two-sided, and a P value < 0.05 was considered statistically significant.

## RESULTS

### Patient characteristics

A total of 2712 RCC patients had cT1a disease, and 121 (4.5%) had pT3a upstaging. After screening, 510 patients with cT1a were finally enrolled in our study including 102 patients in the pT3a upstaging subgroup and 408 patients in the consistent pT1a subgroup. The median age was 53 years (range, 22-83), the median tumor size was 3 cm (range, 0.6-4.0), the median ROD was 1.29 (range, 1.0-3.18), and the median FIB was 2.89 g/L (range, 1.44-6.59). In the pT3a upstaging subgroup, 50 (49.0%) patients had perinephric adipose invasion, 51 (50.0%) patients had sinus fat invasion, 27 (26.5%) patients had segmental renal vein invasion, and 4 patients had pelvicalyceal system invasion. There were 27 patients who had more than two types of pathological invasion (Table-1). The whole population was split into a training cohort (255 patients) and a validation cohort (255 patients) (Table-2).

**Table 1:** Baseline characteristics of pT3a upstaged patients and pT1a patients.

		Overall (n=510)	pT3a (n=102)	pT1a (n=408)	P-value
**Age (years)**					0.009
	Mean (SD)	53.4 (11.3)	57.9 (11.0)	52.3 (11.1)	
	Median [Min, Max]	53 (22, 83)	57 (30, 80)	52 (22, 83)	
**Sex**					0.901
	Male	323 (63.3%)	64 (62.7%)	259 (63.4%)	
	Female	187 (36.7%)	38 (37.3%)	149 (36.6%)	
**BMI (kg/m2)**					0.886
	< 25	214(42.0%)	42 (41.2%)	172 (42.2%)	
	> 25	296 (58.0%)	60 (58.8%)	236 (57.8%)	
**Size (cm)**					< 0.001
	Mean (SD)	2.83 (0.83)	3.19 (0.68)	2.75 (0.85)	
	Median [Min, Max]	3.00 [0.60, 4.00]	3.35 [1.30, 4.00]	2.90 [0.60, 4.00]	
**Location**					0.368
	Upper	125 (24.5%)	21 (20.6%)	104 (25.5%)	
	Middle	245 (48.0%)	58 (56.9%)	187 (45.8%)	
	Lower	140 (27.5%)	23 (22.5%)	117 (28.7%)	
**R.E.N.A.L Score**					0.263
	Low	168 (32.9%)	29 (28.4%)	139 (34.1%)	
	Moderate	285 (55.9%)	57 (55.9%)	228 (55.9%)	
	High	57 (11.2%)	16 (15.7%)	41 (10.0%)	
**ROD**					0.007
	Mean (SD)	1.37 (0.31)	1.46 (0.31)	1.34 (0.30)	
	Median [Min, Max]	1.29 [1.00, 3.18]	1.40 [1.04, 2.50]	1.25 [1.00, 3.18]	
**Type of nephrectomy**					
	Partial	136 (26.7%)	27 (26.5%)	109 (26.7%)	1
	Radical	374 (73.3%)	75 (73.5%)	299 (73.3%)	
**Pathology**					1
	Clear cell carcinoma	420 (82.4%)	84 (82.4%)	336 (82.4%)	
	Non-clear cell carcinoma	90 (17.6%)	18 (17.6%)	72 (17.6%)	
**Etiology of pT3a Upstaging**					
	Perinephric Adipose		50 (49.0%)	NA	
	Renal Sinus Fat Invasion		51 (50.0%)	NA	
	Pelvicalyceal system		4 (3.9%)	NA	
	Segmental Renal Vein		27 (26.5%)	NA	
**FIB (g/L)**					0.001
	Mean (SD)	2.91 (0.64)	3.12 (0.70)	2.86 (0.62)	
	Median [Min, Max]	2.89 [1.44, 6.59]	3.04 [1.44, 4.78]	2.85 [1.50, 6.59]	
**MPV**					0.52
	Mean (SD)	25.3 (34.6)	23.5 (33.0)	25.7 (35.0)	
	Median [Min, Max]	10.7 [1.03, 142]	10.5 [8.39, 131]	10.8 [1.03, 142]	
**AST/ALT**					0.799
	Mean (SD)	1.01 (0.371)	1.01 (0.319)	1.01 (0.384)	
	Median [Min, Max]	0.94 [0.10, 2.80]	0.95 [0.34, 2.00]	0.94 [0.10, 2.80]	

**ALT** = alanine aminotransferase; **AST** = aspartate aminotransferase; **BMI** = body mass index; **FIB** = fibrinogen; **PV** = mean platelet volume; **ROD** = the ratio of the tumor maximum and minimum diameter

**Table 2:** Characteristics of the training and validation cohorts.

		Training (n=255)	Validation (n=255)	P-value
**Age (years)**				0.697
	Mean (SD)	53.3 (11.1)	53.5 (11.5)	
	Median [Min, Max]	53 (22, 80)	53 (25, 83)	
**Sex**				0.849
	Male	172 (67.5%)	175 (68.6%)	
	Female	83 (32.5%)	80 (31.4%)	
**Size (cm)**				0.534
	Mean (SD)	2.83 (0.835)	2.84 (0.834)	
	Median [Min, Max]	3.00 [0.60, 4.00]	3.00 [1.00, 4.00]	
**Location**				0.217
	Upper	56 (22.0%)	69 (27.1%)	
	Middle	134 (52.5%)	111 (43.5%)	
	Lower	65 (25.5%)	75 (29.4%)	
**R.E.N.A.L Score**				1.000
	Low	84 (32.9%)	84 (32.9%)	
	Moderate	143 (56.1%)	142 (55.7%)	
	High	28 (11.0%)	29 (11.4%)	
**ROD**				0.360
	Mean (SD)	1.37 (0.31)	1.36 (0.30)	
	Median [Min, Max]	1.32 [1.00, 3.18]	1.27 [1.00, 2.67]	
**FIB (g/L)**				0.262
	Mean (SD)	2.91 (0.64)	2.92 (0.65)	
	Median [Min, Max]	2.88 [1.44, 4.90]	2.89 [1.50, 6.59]	
**MPV**				0.451
	Mean (SD)	23.5 (32.8)	27.1 (36.3)	
	Median [Min, Max]	10.7 [1.03, 131]	10.8 [7.98, 142]	
**AST/ALT**				0.564
	Mean (SD)	1.01 (0.348)	1.01 (0.394)	
	Median [Min, Max]	0.950 [0.38, 2.22]	0.931 [0.10, 2.80]	
**pT3a**				0.319
	No	209 (82.0%)	199 (78.0%)	
	Yes	46 (18.0%)	56 (22.0%)	

**ALT** = alanine aminotransferase; **AST** = aspartate aminotransferase; **FIB** = fibrinogen; **MPV** = mean platelet volume; **ROD** = the ratio of the tumor maximum and minimum diameter

Risk factors screened from preoperative blood indexes and independent diagnostic factors in the training cohort

Using LASSO regression analysis, the most important risk factor from 40 peripheral blood indicators before surgery was FIB (Supplementary Figure-S1).

**Figure 1:**
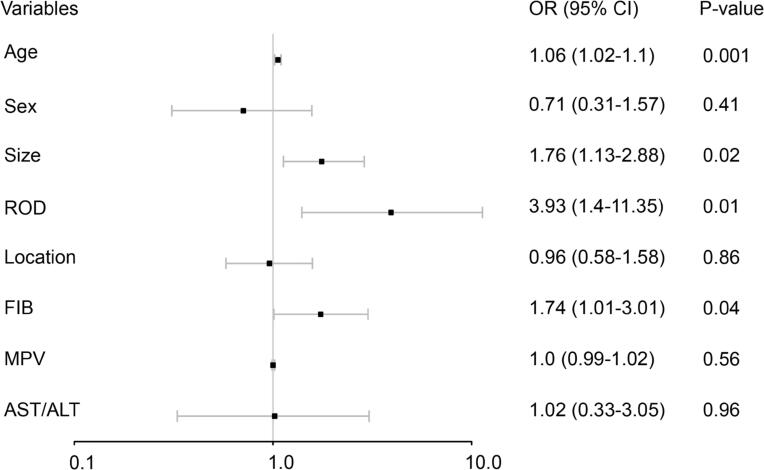
Forest plots of multivariate logistic analysis in the training cohort.

The multivariate logistic regression analysis showed that a larger tumor size (odds ratio: 1.76, 95% CI: 1.13-2.88, P<0.001) was an independent risk factor for upstaging, as well as older age (OR: 1.06, 95% CI: 1.02-1.1, P = 0.004), larger ROD (OR: 3.93, 95% CI: 1.4-11.35, P=0.03) and high levels of FIB (OR: 1.74, 95% CI: 1.01-3.01, P = 0.01). Neither the MPV (OR: 1.0, 95% CI: 0.99-1.02, P = 0.29) nor the AST/ALT ratio (OR: 1.02, 95% CI: 0.33-3.05, P = 0.33) were independent risk factors (Figure-1).

Development of a nomogram of a diagnostic ARFS model for pathological T3a upstaging

**Figure 2:**
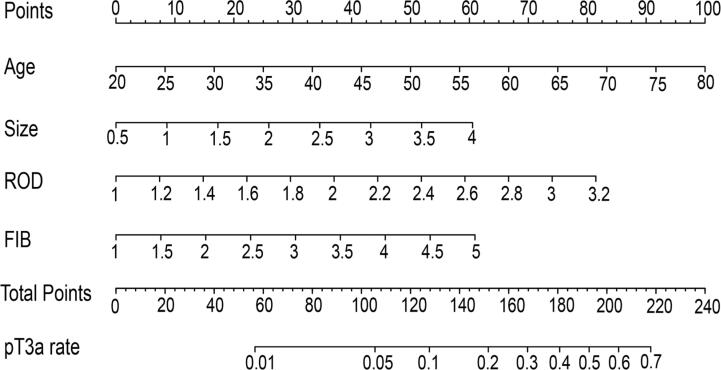
Construction of the nomogram for the ARFS model combining age, the ratio of the tumor maximum and minimum diameter (ROD), fibrinogen (FIB), and tumor size.

As shown in Figure-2, a diagnostic ARFS model nomogram that included age, ROD, FIB, and tumor size for pT3a upstaging was established. The C-index for the prediction of RCC pathological upstaging from cT1a to pT3a in the training cohort was 0.756 (95% CI, 0.681-0.831).

Validation of the predictive accuracy of the ARFS model for pathological T3a upstaging

**Figure 3:**
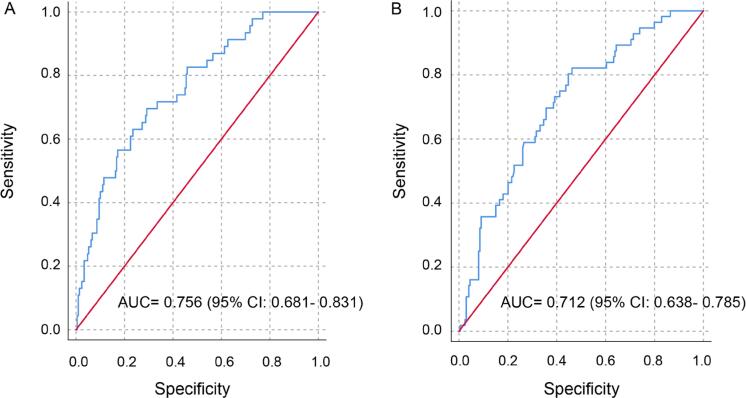
Receiver operating characteristic curves for the predictive ARFS model in the training cohort (A) and in the validation cohort (B).

In the validation cohort, the C-index of the nomogram for predicting pT3a upstaging was 0.712 (95% CI, 0.638-0.785), which was also confirmed in receiver operating curve analysis (Figure-3). This was consistent with the results obtained from the training cohort. This result again suggested that the nomogram model was useful for predicting pT3a upstaging from cT1a in patients with renal cell carcinoma.

## DISCUSSION

In this study, we sought to determine predictors for RCC upstaging from cT1a to pT3a and built a predictive model that could guide surgeons to perform PN or RN in patients with clinical T1a RCC. With regard to the individual variables, age, ROD, FIB, and tumor size appeared to be associated with upstaging risk. The C-index for the nomogram was 0.712 (95% CI, 0.638-0.785). Compared with other studies (20, 21), the advantage of our study was that the ARFS nomogram was more objective and could be easily calculated with 4 preoperative quantitative risk factors.

The clinical benefits of PN and RN in pT3a RCC remain highly debated. Several previous retrospective studies have indicated that PN had worse oncological outcomes in upstaging patients (9, 12). Alvim et al. did not find a significant difference in complication rate or oncological survival between planned PN and RN for pT3a RCC (22). Veccia et al. conducted a systematic review and meta-analysis on upstaging to pT3a RCC patients and demonstrated that five-year recurrence-free survival was worse in the upstaged group (p = 0.02) perhaps due to positive surgical margins. However, there is very limited evidence regarding whether RN would be better than PN in these cases (13). Recently, a newly published meta-analysis involving 12 studies on pT3a RCC indicated that there were no significant differences between PN and RN in terms of the operative time, surgical complications, or oncological survival (14). In summary, PN might be a suitable choice for upstaging patients, but close attention should be given to avoid positive surgical margins. On the other hand, predicting pT3a RCC by a nomogram can assist urologists in screening localized cT1 RCC patients as perinephric fat, sinus fat or segmental renal vein invasion might weaken the local control efficacy of ablation therapies (23, 24).Our ARFS nomogram may benefit therapeutic decisions regarding ablation therapy, especially for cT1a patients.

Some reports have demonstrated that older age and increased tumor size are independently associated with renal cell carcinoma upstaging from cT1 to pT3a (15, 17, 25). The findings from these reports are consistent with the results of our research in which larger tumor size (OR: 1.76) and increasing age (OR: 1.06) were independently associated with pT3a upstaging.

On the three-dimensional plane of the tumor, the longest and shortest maximum diameters could be calculated, and we defined a parameter factor as the ROD in previous research (19). As an innovative predictor, the ROD contained the morphological characteristics of the tumor and may reflect the polycentric developmental characteristics and aggressive proliferation of RCC. Recently, Teishima et al reported the impact of the radiological morphology of RCC cT1 on the prediction of pT3 upstaging. They classified the tumor into 3 types: round, lobular or irregular, and their results suggested that an irregular radiological morphology could predict the pathological upstaging to T3a (26). We depicted the morphology as a quantitative value that was more objective and easily calculated.

In a previous study, we found that the preoperative FIB level was positively correlated with the circulating tumor cell (CTC) count and that FIB was an independent prognostic marker of RCC (27, 28). Another meta-analysis demonstrated that elevated pretreatment plasma fibrinogen is associated with poorer survival in renal cell carcinoma (OS: HR=2.13, CSS: HR=2.99) (29).

In contrast to other studies, our study focused on cT1a upstaging. According to the ARFS model, we should pay attention to the resection of perirenal fat and renal parenchyma during PN for patients with a higher risk of upstaging. Although the relationships between positive surgical margins and the recurrence rate and survival are controversial (9, 30-32), it is better to perform RN or complete resection of tumor by RN for possible T3 RCC patients. If PN is the absolute indication and the AFRS nomogram indicates a higher upstaging risk for some RCC patients, intraoperative ultrasound might be necessary to decrease the risk of positive surgical margins (33).

The limitations of this study are as follows: first, there is inherent bias associated with its retrospective design, and it was a study with a large time span. Second, this is a single-institutional analysis. In the future, we hope to carry out multicenter and prospective studies to further verify the predictive performance of the model.

## CONCLUSIONS

In summary, age, the ratio of the tumor maximum and minimum diameter, fibrinogen, and tumor size were independent risk factors for upstaging. The novel model that combines these four factors could aid in predicting pT3a upstaging in patients with cT1a RCC. Large-scale multicenter studies may be needed to confirm this model in the future.

## Figures and Tables

**Supplementary Figure S1:**
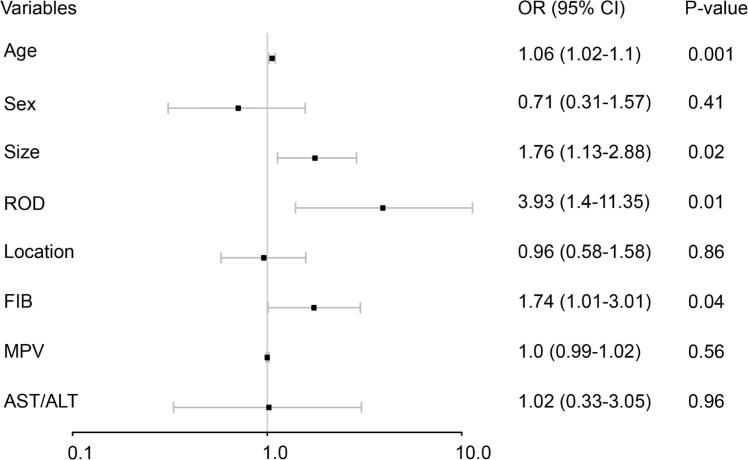
Least absolute shrinkage and selection operator regression analysis was used to identify the top risk factor, fibrinogen (FIB), which was screened from 40 peripheral blood indicators.

## ABBREVIATIONS

ALT: alanine aminotransferase
AST: aspartate aminotransferase
CAMS: Chinese Academy of Medical Sciences
C-index: concordance index
CT: computer tomography
cT1a: clinical T1a
FIB: fibrinogen
LASSO: least absolute shrinkage and selection operator
MR: magnetic resonance
MVP: mean platelet volume
OR: odds ratio
PN: partial nephrectomy
pT1a: pathological T1a
pT3a: pathological T3a
RCC: Renal cell carcinoma
RN: radical nephrectomy
ROD: the ratio of the tumor maximum and minimum diameter

## APPENDIX

### The 40 blood indexes

WBC, white blood cell count; Hb, haemoglobin; PLT, platelet count; PDW, platelet distribution width; PCT, platelet haematocrit, HCT, haematocrit; NEUT, neutrophil count; NETU%, neutrophil percentage; LYMPH, lymphocyte count; MONO, monocyte count; MONO%, monocyte percentage; NLR, neutrophil-lymphocyte ratio; EOS, eosinophils count; BAS, basophils count; AST, aspartate aminotransferase; ALT, alanine aminotransferase; MPV, mean platelet volume; ALP, alkaline phosphatase; GGT, glutamyl transferase; LDH, lactate dehydrogenase; TBIL, total bilirubin; DBIL;TP, total protein; Alb, albumin; URIC, uric acid; BUN, blood urea nitrogen; CRE, creatinine; IgA, immunoglobulin A; IgG, immunoglobulin G; IgM, immunoglobulin M; CRP, C-reactive protein; APTT, activated partial thromboplastin time; FIB, fibrinogen; PLR, platelet-lymphocyte ratio; LMR, lymphocyte-monocyte ratio; potassium kalium; sodium; chlorine; calcium.
